# Maternal Pertussis Immunization and Immunoglobulin G Levels in Early- to Late-Term and Preterm Infants

**DOI:** 10.1001/jamanetworkopen.2024.24608

**Published:** 2024-07-30

**Authors:** Maarten M. Immink, Mireille N. Bekker, Hester E. de Melker, Gerco den Hartog, Nynke Y. Rots, Pieter G. M. van Gageldonk, Floris Groenendaal, Elisabeth A. M. Sanders, Nicoline A. T. van der Maas

**Affiliations:** 1 Centre for Infectious Disease Control, National Institute for Public Health and the Environment, Bilthoven, the Netherlands; 2 Department of Obstetrics, Wilhelmina Children’s Hospital, University Medical Center Utrecht and Utrecht University, Utrecht, the Netherlands; 3 Laboratory of Medical Immunology, Radboud University Medical Center, Nijmegen, the Netherlands; 4 Department of Neonatology, Wilhelmina Children’s Hospital, University Medical Center Utrecht and Utrecht University, Utrecht, the Netherlands; 5 Department of Pediatric Immunology and Infectious Diseases, Wilhelmina Children’s Hospital, University Medical Center Utrecht and Utrecht University, Utrecht, the Netherlands

## Abstract

**Importance:**

Maternal tetanus, diphtheria, and acellular pertussis (Tdap) vaccination protects newborns against severe pertussis. Data on transplacental antibody transfer on Tdap vaccination before 24 weeks’ gestation remain scarce and are particularly relevant for preterm infants to increase the time interval for maternal antibody transfer.

**Objective:**

To assess noninferiority of anti–pertussis toxin (anti-PT) immunoglobulin G (IgG) antibody levels at age 2 months in early- to late-term infants following Tdap vaccination between 20 0/7 and 24 0/7 weeks’ gestation compared with 30 0/7 and 33 0/7 weeks’ gestation and compared with preterm infants.

**Design, Setting, and Participants:**

This prospective, multicenter cohort study included pregnant women aged 18 years or older in birthing centers and hospitals in the Netherlands between August 2019 and November 2021 who received Tdap vaccination between 20 0/7 and 24 0/7 weeks’ gestation. Women with imminent premature birth were recruited if they had received maternal Tdap vaccination between 20 and 24 weeks’ gestation. Blood samples were collected from mothers at delivery, from the umbilical cord, and from infants at age 2 months. Data from infants’ blood samples at age 2 months were compared with a reference cohort (recruited between January 2014 and February 2016) of early- to late-term infants of the same age whose mothers had received Tdap vaccination between 30 0/7 and 33 0/7 weeks’ gestation.

**Exposure:**

Maternal Tdap vaccination between 20 0/7 and 24 0/7 weeks’ gestation or 30 0/7 and 33 0/7 weeks’ gestation.

**Main Outcomes and Measures:**

The primary outcome was the geometric mean concentration (GMC) of anti-PT IgG antibodies in early- to late-term infants (≥37 0/7 weeks’ gestation) at age 2 months, comparing maternal Tdap vaccination between 20 0/7 and 24 0/7 weeks’ vs 30 0/7 and 33 0/7 weeks’ gestation (reference cohort). Anti-PT GMC in 2-month-old infants born preterm (<35 0/7 weeks’ gestation) compared with early- to late-term infants after maternal Tdap vaccination between 20 and 24 weeks’ gestation was a secondary outcome.

**Results:**

In total, 221 women who delivered 239 offspring were enrolled in the study; 66 early- to late-term infants (median gestational age [GA], 40.6 weeks [IQR, 39.8-41.0 weeks]; 38 [57.6%] male) and 73 preterm infants (median GA, 32.1 weeks [IQR, 29.5-33.0 weeks]; 42 [54.5%] female) had blood samples collected at 2 months of age. Anti-PT GMC was 14.7 IU/mL (95% CI, 10.6-20.4 IU/mL) in early- to late-term infants following maternal Tdap vaccination between 20 0/7 and 24 0/7 weeks’ gestation compared with 27.3 IU/mL (95% CI, 20.1-37.1 IU/mL) in 55 infants in the reference group (median GA, 40.3 [IQR, 39.1-41.0]; 33 [60.0%] female). The mean anti-PT GMC in preterm infants in the study group was 11.2 IU/mL (95% CI, 8.1-15.3 IU/mL) (*P* = .23 compared with early- to late-term infants).

**Conclusions and Relevance:**

In this cohort study, 2-month-old preterm and early- to late-term infants showed significantly lower anti-PT antibody levels following maternal Tdap vaccination between 20 0/7 and 24 0/7 weeks’ gestation compared with 30 0/7 and 33 0/7 weeks’ gestation; preterm and early- to late-term infants had similar anti-PT antibody levels, but both groups showed significantly lower antibody levels compared with the reference group. Epidemiological research should investigate whether maternal Tdap vaccination before 24 weeks’ gestation provides sufficient protection against clinical pertussis, particularly in preterm infants, as long as no correlate of protection is available.

## Key Points

**Question:**

Is maternal tetanus, diphtheria, and acellular pertussis (Tdap) vaccination between 20 and 24 weeks’ gestation noninferior to vaccination between 30 and 33 weeks gestation with respect to maternal-derived anti–pertussis toxin (anti-PT) antibody levels in early- to late-term (≥37 weeks) and preterm (<35 weeks) infants at age 2 months?

**Findings:**

In this cohort study of 221 women and 239 offspring, anti-PT antibody levels following maternal Tdap vaccination between 20 and 24 weeks’ gestation in 66 early- to late-term infants were significantly lower compared with levels in 55 early- to late-term infants (reference) after maternal vaccination from 30 to 33 weeks’ gestation. The anti-PT geometric mean concentration in 73 preterm infants was substantially lower than in the reference group despite a similar median time from maternal vaccination to delivery.

**Meaning:**

The findings suggest that maternal Tdap immunization before 24 weeks’ gestation is associated with less protection against pertussis among early- to late-term and preterm infants.

## Teaser

This cohort study compares maternal-derived anti–pertussis toxin immunoglobulin G (IgG) antibody levels in 2-month-old early- to late-term and preterm infants whose mothers received tetanus, diphtheria, and acellular pertussis vaccination between 20 and 24 weeks’ vs 30 and 33 weeks’ gestation.

## Introduction

According to the World Health Organization (WHO), 81% of infants worldwide (105 million) received 3 doses of a diphtheria, tetanus, and pertussis vaccine in 2021, protecting them against vaccine-preventable diseases that may cause serious, even fatal, illness and disability.^1^ Despite high vaccine coverage, pertussis remains endemic in many countries. Newborns and infants too young to be fully vaccinated are at the highest risk of severe complications.^2^ To protect newborns and infants in the first months of life, maternal vaccination with a tetanus, diphtheria, and acellular pertussis (Tdap) vaccine from 20 weeks’ gestation onward has been offered to all pregnant women in the Netherlands since December 2019. Infant diphtheria, tetanus, and pertussis (DTaP), inactivated poliovirus (IPV), *Haemophilus influenzae* type b, and hepatitis B vaccinations are given at 3, 5, and 11 months of age (2 + 1 dose schedule) for protection against pertussis provided that the mother received Tdap vaccination during pregnancy. An extra vaccination at 2 months of age (3 + 1 dose schedule) after maternal Tdap vaccination is advised if an infant is born before 37 weeks’ gestation or if the time interval between maternal vaccination and delivery is shorter than 2 weeks, since transfer of immunity against pertussis on maternal Tdap vaccination may be insufficient.

During pregnancy, maternal immunoglobulin G (IgG) antibodies are actively transferred across the placenta, mediated by the neonatal Fc receptor expressed on syncytiotrophoblast cells. This saturable process initiates at approximately 13 to 17 weeks’ gestation and increases throughout gestation. Around 33 to 36 weeks’ gestation, fetal IgG antibody levels exceed maternal IgG serum levels and increase to 150% of maternal levels near the due delivery date.^3^ Tdap vaccination in the third trimester enhances maternal antipertussis IgG antibody levels in newborns.^4,5,6,7^ Maternal Tdap vaccination was reported to prevent 70% to 90% of clinically confirmed pertussis cases and about 90.5% of pertussis hospitalizations in newborns and infants younger than 3 months of age in the UK from 2013 to 2018.^8,9^

There is no consensus on the optimal timing of maternal Tdap vaccination to achieve the highest antibody transfer. Most studies suggest that Tdap vaccination early in the third trimester results in the highest anti–pertussis toxin (anti-PT) IgG antibody levels at birth,^4,5,6,7^ while a Swiss study favored second-trimester vaccination, potentially due to a longer time interval between Tdap vaccination and delivery.^10,11^ Recently, it was estimated that a period of 7.5 weeks or more before delivery optimizes antibody transfer.^5^ Tdap vaccination before 24 weeks’ gestation may therefore be particularly relevant for preterm offspring, the group most vulnerable for severe pertussis. Preterm infants have a hospitalization rate for pertussis that is 1.5-times higher than predicted based on the total proportion of infants in the national UK birth cohort.^12^ Offering maternal Tdap vaccination from 20 weeks’ gestation also widens the opportunity for pregnant women to receive the vaccine, but few studies have reported antibody levels after maternal Tdap vaccination at or before 24 weeks’ gestation or in preterm infants.^6,11^ These studies had insufficient power to draw firm conclusions.

In this study, pertussis-specific IgG antibody levels after maternal Tdap vaccination between 20 0/7 and 24 0/7 weeks’ gestation were evaluated in early- to late-term (hereafter, *term*) and preterm offspring with follow-up until 2 months of age. We primarily assessed whether maternal Tdap vaccination between 20 0/7 and 24 0/7 weeks’ gestation would be associated with similar anti-PT antibody levels in term infants at 2 months of age compared with maternal Tdap vaccination between 30 0/7 and 33 0/7 weeks’ gestation. Therefore, data were compared with those from a reference study (recruitment between January 2014 and February 2016) including 55 term infants following maternal Tdap vaccination between 30 0/7 and 33 0/7 weeks’ gestation.^13^ Additionally, we compared antibody levels in term and preterm infants following maternal Tdap vaccination between 20 0/7 and 24 0/7 weeks’ gestation.

## Methods

### Study Participants

In this prospective, multicenter cohort study, antenatal care practitioners working in birthing centers or hospitals recruited pregnant women aged 18 years or older between August 2019 and November 2021. The study design and procedures were previously described.^14^ In brief, women were included through 2 recruitment routes; from August 2019, healthy pregnant women were invited to participate and received Tdap vaccination between 20 0/7 and 24 0/7 weeks’ gestation as part of the study. In addition, after 2019, once the Dutch National Immunisation Programme (NIP) offered Tdap vaccination to all pregnant women from 20 weeks’ gestation onward, women with imminent preterm labor were recruited on presentation at the hospital provided that they received Tdap vaccination between 20 0/7 and 24 0/7 weeks’ gestation. These women were vaccinated through the NIP, unrelated to this study but with the same Tdap vaccine as used in the study. Women were excluded if they had received Tdap vaccination within the past 2 years or if there was a known or suspected underlying condition that could interfere with study results. Other exclusion criteria were previously described.^14^ Mother-infant pairs were followed up until 2 months after delivery. Data on *Bordetella pertussis*–specific IgG antibodies from mother-infant pairs in the study were compared with data from the reference study performed between January 2014 and February 2016 that comprised term infants at age 2 months after maternal Tdap vaccination between 30 0/7 and 33 0/7 weeks’ gestation.^13^ Both studies used identical vaccines and study procedures for collection and timing of collection of blood samples. Laboratory procedures were performed in the same laboratory using identical procedures. This study was conducted in accordance with the Declaration of Helsinki^15^ and approved by the Central Committee on Research Involving Human Subjects in the Netherlands. Oral and written informed consent was obtained from parents or legal guardians. The study followed the Strengthening the Reporting of Observational Studies in Epidemiology (STROBE) reporting guideline.

### Maternal Vaccine

Pregnant women received a Tdap vaccine (Boostrix) containing adsorbed *B pertussis* antigens (ie, inactivated PT, filamentous hemagglutinin [FHA], pertactin [Prn], diphtheria toxoid [DT], and tetanus toxoid [TT]).^16^ The Tdap vaccine was administered as a single 0.5-mL intramuscular injection in the deltoid muscle.

### Blood Sampling

Finger-stick blood samples (≤300 μL) were collected from mothers within 24 hours after delivery. Umbilical cord blood samples (≤2 mL) were collected at delivery, and heel-stick blood samples from infants (≤300 μL) were collected during home visits before primary vaccination at age 2 months (±5 days). For preterm infants, who often start receiving vaccinations between 6 and 9 weeks in the Netherlands, blood samples were collected before the first vaccination. Serum samples were stored at −20 °C awaiting analyses.

### Laboratory Analyses

Immunoglobulin G antibody concentrations against PT, FHA, Prn, DT, and TT were measured by bead-based fluorescent multiplex immunoassay using Luminex xMAP technology (ThermoFisher Scientific), as previously described.^17^ For the *B pertussis* antigens, the assay was calibrated against the WHO international standard for pertussis antiserum (serum reference 06/140), interpolated using a 5-parameter fit, and expressed in international units (IU/mL).

### Statistical Analysis

Anti-PT IgG antibody levels following maternal Tdap vaccination are associated with prevention of clinical pertussis.^18^ Our primary outcome was to assess noninferiority of anti-PT antibody levels in term infants at 2 months of age following maternal Tdap vaccination between 20 0/7 and 24 0/7 weeks’ gestation compared with Tdap vaccination between 30 0/7 and 33 0/7 weeks’ gestation (reference cohort). The lower limit of the 95% CI of the geometric mean concentration ratio (GMR) between the main and the reference cohorts was set at 0.5 or greater for noninferiority. Secondary outcomes were the geometric mean concentration (GMC) of PT IgG levels in preterm infants at 2 months’ postnatal age after maternal Tdap vaccination between 20 0/7 and 24 0/7 weeks’ gestation compared with the term cohort after maternal Tdap vaccination between 20 and 24 weeks’ gestation and the IgG antibody levels against all Tdap vaccine antigens (ie, PT, FHA, Prn, DT, and TT) in blood samples from infants at 2 months of age, the umbilical cord, and mothers at delivery among term and preterm mother-infant pairs (eFigure in Supplement 1).

To assess our primary research question and allow 80% power and an α of 5%, 58 term and 54 preterm mother-infant pairs were required.^14^ We aimed for inclusion of 60 pairs in each group to allow loss to follow-up regarding the available blood samples.

For the scope of this study, we defined preterm as birth between 24 0/7 and 34 6/7 weeks’ gestation since offspring antibody levels are expected to exceed maternal antibody levels at the end of this time window, and these late-preterm offspring may therefore resemble offspring born at full term regarding transplacental antibody transfer.^3^ Term birth was defined as 37 0/7 or more weeks’ gestation.

Comparison of baseline characteristics was done using either *t*, Mann-Whitney *U*, or Fisher exact test. The IgG-antibody concentrations against all antigens were log-transformed and computed into GMCs with corresponding 95% CIs. In all groups, including the reference cohort,^13^ GMCs at different time points were assessed using generalized estimating equation models with a gaussian distribution with identity link function. An exchangeable correlation structure enabled adjustment for similarities in antibody levels among siblings who were twins or triplets. No additional adjustment was applied. The GMRs were calculated from the GMCs within different groups and expressed with 95% CIs. R, version 2023.03.1 (R Project for Statistical Computing) was used with the geepack package for analyses.^19^ Findings were based on available data, and missing data were handled by complete participant analyses. Two-sided *P* < .05 was considered significant.

## Results

**Figure 1.**
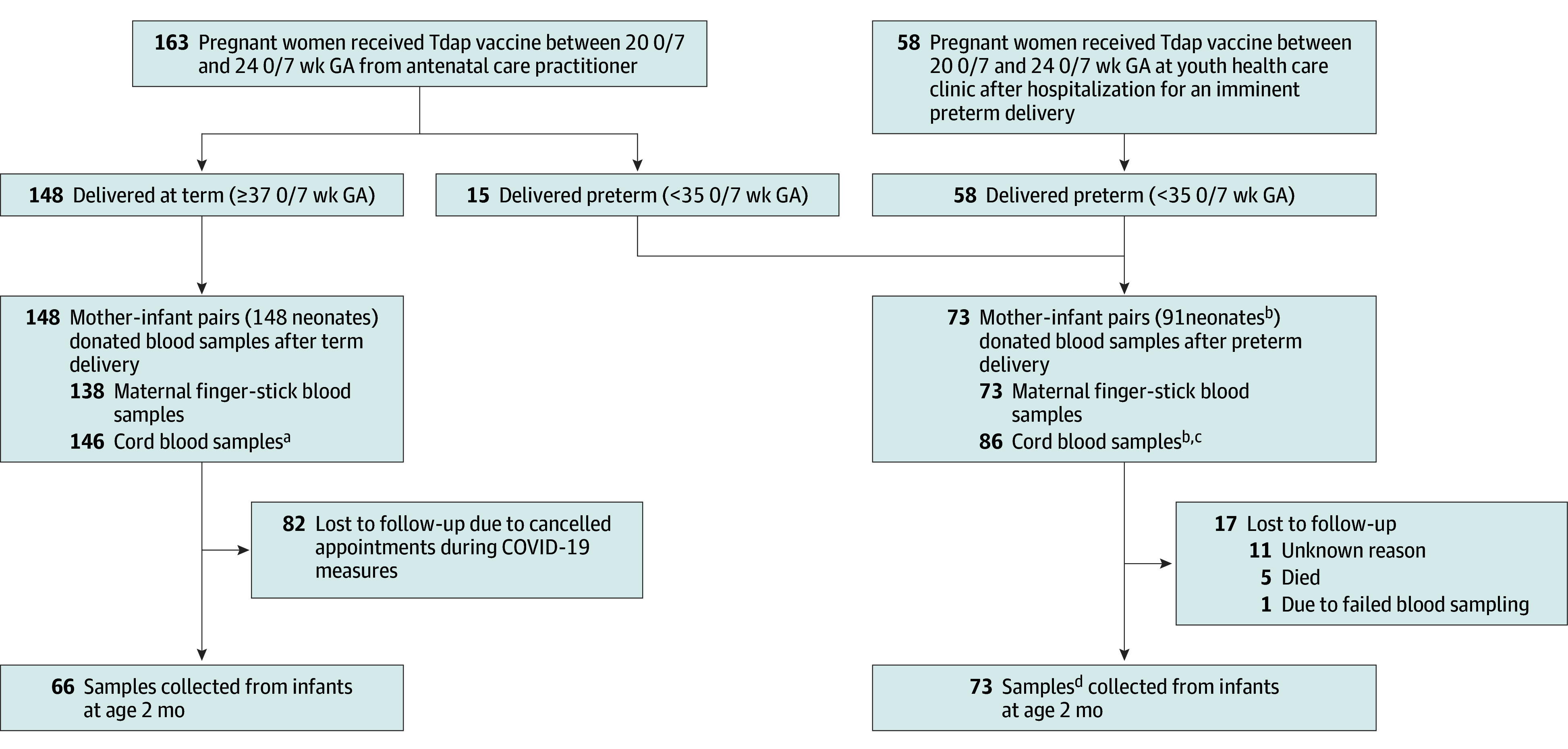
Flowchart of Study Procedures *Term* includes early- to late-term births (≥37 0/7 weeks’ gestation). GA indicates gestation; Tdap, tetanus, diphtheria, and acellular pertussis. ^a^Including 14 pairs of twins and 2 sets of triplets, among whom 1 sample was not collected due to perinatal death. ^b^Two infants had a sample obtained at 2 months but no umbilical cord blood sample. ^c^Four infants had a sample obtained at 2 months but no umbilical cord blood sample. ^d^Seventy-three infants from 60 mothers had a blood sample obtained at 2 months of age.

In total, 221 pregnant women who received second-trimester Tdap vaccination were included. They delivered 239 offspring, of whom 148 (61.9%) were term and 91 (38.1%) were preterm. The preterm offspring included 14 pairs of twins and 2 sets of triplets. All 148 term offspring (range, 37 0/7-42 0/7 weeks’ gestation) were singletons; 66 of these (28 [42.4%] female; 38 [57.6%] male) had a blood sample collected at 2 months of age. The 91 preterm offspring (range, 25 2/7-34 6/7 weeks’ gestation) were born to 73 mothers, and 73 of these offspring (42 [54.5%] female; 35 [45.5%] male) had a blood sample obtained at age 2 months (Figure 1). Detailed demographics of study and reference mother-infant pairs are shown in Table 1. The median gestational age (GA) at birth was not significantly different between term infants in the study group (40.6 weeks [IQR, 39.8-41.0 weeks]) and the reference group of 55 infants (33 [60.0%] female; 22 [40.0%] male; median GA, 40.3 weeks [IQR, 39.1-41.0 weeks]). Median GA was significantly different for preterm offspring (32.1 weeks [IQR, 29.5-33.0 weeks]) compared with term offspring in both the study cohort and the reference cohort. The media GA at maternal Tdap vaccination in the study groups of term and preterm mother-infant pairs (22.0 weeks [IQR, 20.9-23.1 weeks] and 22.9 weeks [IQR, 22.0-23.4 weeks], respectively) was significantly different from the median GA in the term mother-infant pairs in the reference cohort (31.1 weeks [IQR, 30.5-31.7 weeks]). The median time interval between maternal Tdap vaccination and delivery was 18.3 weeks (IQR, 17.1-19.7 weeks) for term births and 9.4 weeks (IQR, 6.9-10.7 weeks) for preterm births in the study cohort and 9.0 weeks (IQR, 8.1-9.9 weeks) for term births in the reference cohort (Table 1).^13^

**Table 1.** Baseline Characteristics at the Mother and Infant Level for Preterm and Early- and Full-Term Mother-Infant Pairs^**a**^

Characteristic	Participants, by GA at maternal Tdap vaccination^b^		
	20 0/7-24 0/7 wk		30 0/7-33 0/7 wk, Term birth (n = 55)^d^
	Preterm birth (n = 73)^c^	Term birth (n = 66)^c^	
Maternal age at delivery, mean (SD), y	31.4 (3.8)	31.7 (4.0)	32.6 (3.3)
GA at maternal immunization, median (IQR), wk	22.9 (22.0-23.4)	22.0 (20.9-23.1)	31.1 (30.5-31.7)
Pregnancy duration, wk			
Median (IQR)	32.1 (29.5-33.0)	40.6 (39.8-41.0)	40.3 (39.1-41.0)
25 2/7-27 6/7	13 (17.8)	NA	NA
28 0/7-31 6/7	25 (34.2)	NA	NA
32 0/7-34 6/7	35 (47.9)	NA	NA
37 0/7-39 6/7	NA	21 (31.8)	23 (41.8)
40 0/7-42 0/7	NA	45 (68.2)	32 (58.2)
Interval between maternal immunization and delivery, median (IQR), wk	9.4 (6.9-10.7)	18.3 (17.1-19.7)	9.0 (8.1-9.9)
Multiple pregnancy^e^			
No	45 (78.1)	66 (100)	55 (100)
Twins	13 (19.2)	0	0
Triplets	2 (2.7)	0	0
Infant sex			
Female	42 (54.5)	28 (42.4)	33 (60.0)
Male	35 (45.5)	38 (57.6)	22 (40.0)
Birth weight, mean (SD), g^f^	1631 (499)	3622 (430)	3446 (481)
Birth weight percentile corrected for GA, mean (SD)^f^	38.8 (31.5)	53.1 (27.8)	42.4 (28.0)
Age at blood sample obtainment, mean (SD), d^g^	55.2 (6.2)	61.0 (3.0)	61.4 (2.1)

Abbreviations: GA, gestational age; NA, not applicable; Tdap, tetanus, diphtheria, and acellular pertussis.

^a^ Term birth was defined as GA of 37 0/7 weeks or more and preterm birth as less than 35 0/7 weeks’ gestation.

^b^ Data are presented as number (percentage) of participants unless otherwise indicated.

^c^ In the study cohort, 73 preterm infants born to 60 mothers (due to multiple pregnancies) had a blood sample obtained at 2 months of age, as did 66 term infants.

^d^ In the reference cohort, infants had a blood sample obtained at 2 months of age.

^e^ Seven pairs of dichorionic-diamniotic twins, 4 pairs of monochorionic-diamniotic twins, 2 pairs of monochorionic-monoamniotic twins, and 2 sets of trichorionic-triamniotic triplets. Numbers sum to 60 infants (77 including siblings) for the total number of mother-infant pairs, but only 73 of 77 infants (94.8%) had a blood sample obtained at 2 months of age.

^f^ Birth weight and birth weight percentiles were presented for the firstborn infant only if there were multiple pregnancies.

^g^ Blood samples at age 2 months were obtained as close as possible to infant immunization but may have been obtained earlier than at 2 months of age because, in the Netherlands, routine preterm primary vaccinations are administered between 6 and 9 weeks after birth.

**Figure 2.**
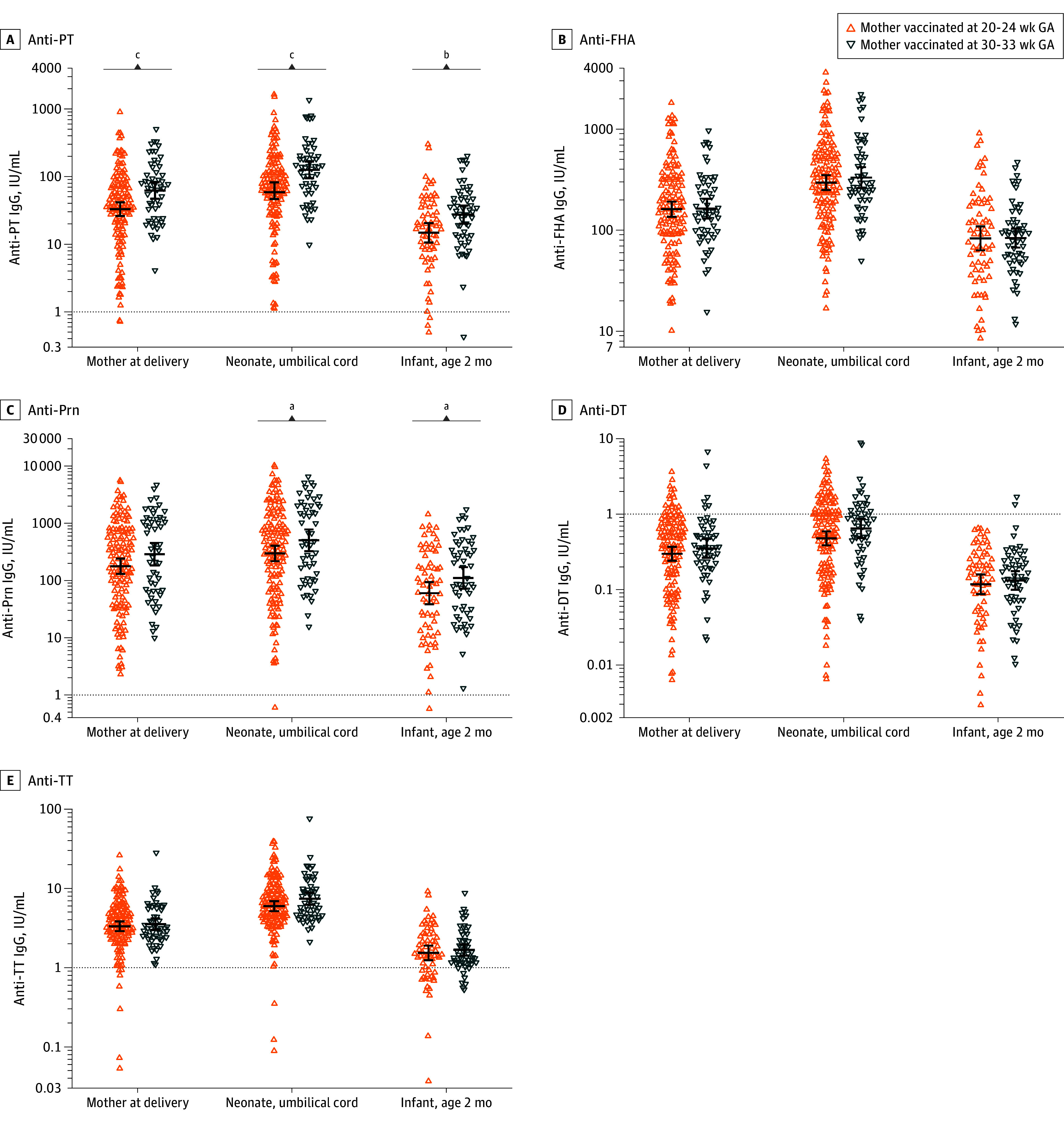
Individual Immunoglobulin G (IgG) Antibody Concentrations and Geometric Mean Concentrations (GMCs) After Second- vs Third-Trimester Tetanus, Diphtheria, and Pertussis Vaccination in Early- and Full-Term Mother-Infant Pairs at Different Time Points Horizontal lines represent GMCs and vertical bars, 95% CIs. DT indicates diphtheria toxoid; FHA, filamentous hemagglutinin; GA, gestation; Prn, pertactin; PT, pertussis toxin; TT, tetanus toxoid. ^a^*P* < .05. ^b^*P* < .01. ^c^*P* < .001.

At 2 months of age in term infants, the anti-PT GMC after maternal Tdap vaccination between 20 0/7 and 24 0/7 weeks’ gestation (14.7 IU/mL; 95% CI, 10.6-20.4 IU/mL) was significantly lower than the GMC in the reference cohort after Tdap vaccination between 30 0/7 and 33 0/7 weeks’ gestation (27.3 IU/mL; 95% CI, 20.1-37.1 IU/mL) (Table 2 and Figure 2). The GMR was 0.54 (95% CI, 0.34-0.85), with the 2.5% bound of the 95% CI at 0.34 (97.5% bound at 0.85) refuting noninferiority requirements.

**Table 2.** GMCs and GMRs in Preterm and Early- and Full-Term Infants With Mothers Vaccinated at 20 0/7 to 24 0/7 Weeks’ Gestation and Term Infants With Mothers Vaccinated at 30 0/7 to 33 0/7 Weeks’ Gestation^a^

Antibody	GMC (95% CI), IU/mL, by GA at maternal Tdap vaccination			GMR (95% CI) at term, study vs reference^b^	*P* value	GMR (95% CI), preterm vs term	*P* value
	20 0/7-24 0/7 wk (study cohort)^b^		30 0/7-33 0/7 wk, Delivery 37 0/7-42 0/7 wk’ gestation, term (reference)^b^				
	Delivery 25 2/7-34 6/7 wk’ gestation, preterm	Delivery between 37 0/7 and 42 0/7 wk’ gestation, term					
**Mothers at delivery**							
Samples, No.	73	138	55	NA	NA	NA	NA
Anti–pertussis toxin	60.4 (44.1-82.7)	32.9 (26.0-41.6)	61.8 (46.8-81.7)	0.53 (0.35-0.80)	<.001	1.84 (1.24-2.72)	.002
Anti–filamentous hemagglutinin	220.5 (171.5-283.5)	161.1 (135.5-191.5)	163.4 (132.5-204.6)	0.99 (0.73-1.34)	.92	1.37 (1.02-1.84)	.04
Antipertactin	203.1 (134.6-306.4)	176.5 (129.9-239.8)	286.0 (182.4-448.3)	0.62 (0.35-1.08)	.08	1.15 (0.69-1.92)	.59
Anti–diphtheria toxoid	0.6 (0.5-0.8)	0.3 (0.2-0.4)	0.4 (0.3-0.5)	0.85 (0.58-1.24)	.35	2.01 (1.50-2.91)	<.001
Anti–tetanus toxoid	5.6 (4.6-6.8)	3.3 (2.9-3.8)	3.5 (3.0-4.2)	0.94 (0.74-1.21)	.59	1.68 (1.31-2.14)	<.001
**Neonatal umbilical cord blood**							
Samples, No.	86	146	54	NA	NA	NA	NA
Anti–pertussis toxin	52.8 (40.7-68.6)	58.6 (46.4-74.2)	125.1 (94.0-166.3)	0.47 (0.31-0.72)	<.001	0.90 (0.63-1.30)	.63
Anti–filamentous hemagglutinin	193.5 (155.2-241.3)	295.2 (249.1-349.9)	330.9 (261.2-419.3)	0.89 (0.65-1.22)	.43	0.66 (0.50-0.87)	.006
Antipertactin	143.7 (97.3-212.4)	295.5 (216.7-402.8)	500.5 (322.5-776.7)	0.59 (0.33-1.05)	.049	0.49 (0.30-0.80)	.02
Anti–diphtheria toxoid	0.5 (0.4-0.7)	0.5 (0.4-0.6)	0.6 (0.5-0.9)	0.74 (0.50-1.09)	.09	1.10 (0.80-1.52)	.40
Anti–tetanus toxoid	5.2 (4.2-6.4)	6.0 (5.2-6.9)	7.4 (6.2-8.8)	0.81 (0.62-1.04)	.06	0.87 (0.68-1.11)	.13
**Infants at 2 mo of age**							
Samples, No.	73	66	55	NA	NA	NA	NA
Anti–pertussis toxin	11.2 (8.1-15.3)	14.7 (10.6-20.4)	27.3 (20.1-37.1)	0.54 (0.34-0.85)	.005	0.76 (0.48-1.20)	.23
Anti–filamentous hemagglutinin	48.8 (37.3-63.8)	83.1 (63.6-109.1)	83.7 (67.4-103.9)	0.99 (0.70-1.42)	.97	0.59 (0.40-0.86)	.009
Antipertactin	38.9 (24.7-61.5)	59.8 (38.4-93.0)	110.3 (71.6-170.0)	0.54 (0.29-1.01)	.045	0.65 (0.35-1.23)	.25
Anti–diphtheria toxoid	0.1 (0.1-0.1)	0.1 (0.1-0.2)	0.1 (0.1-0.2)	0.89 (0.59-1.35)	.57	0.93 (0.63-1.37)	.73
Anti–tetanus toxoid	1.2 (1.0-1.5)	1.5 (1.2-1.9)	1.7 (1.4-2.0)	0.92 (0.70-1.21)	.52	0.78 (0.57-1.06)	.03

Abbreviations: GMC, geometric mean concentration; GMR, geometric mean concentration ratio; NA, not applicable.

^a^ Term birth was defined as a gestational age of 37 0/7 weeks or more and preterm birth as less than 35 0/7 weeks’ gestation.

^b^ Women vaccinated between 20 0/7 and 24 0/7 weeks’ gestation who delivered at term or preterm were included in the general cohort; women vaccinated between 30 0/7 and 33 0/7 weeks’ gestation who delivered at term were included as the reference cohort.

**Figure 3.**
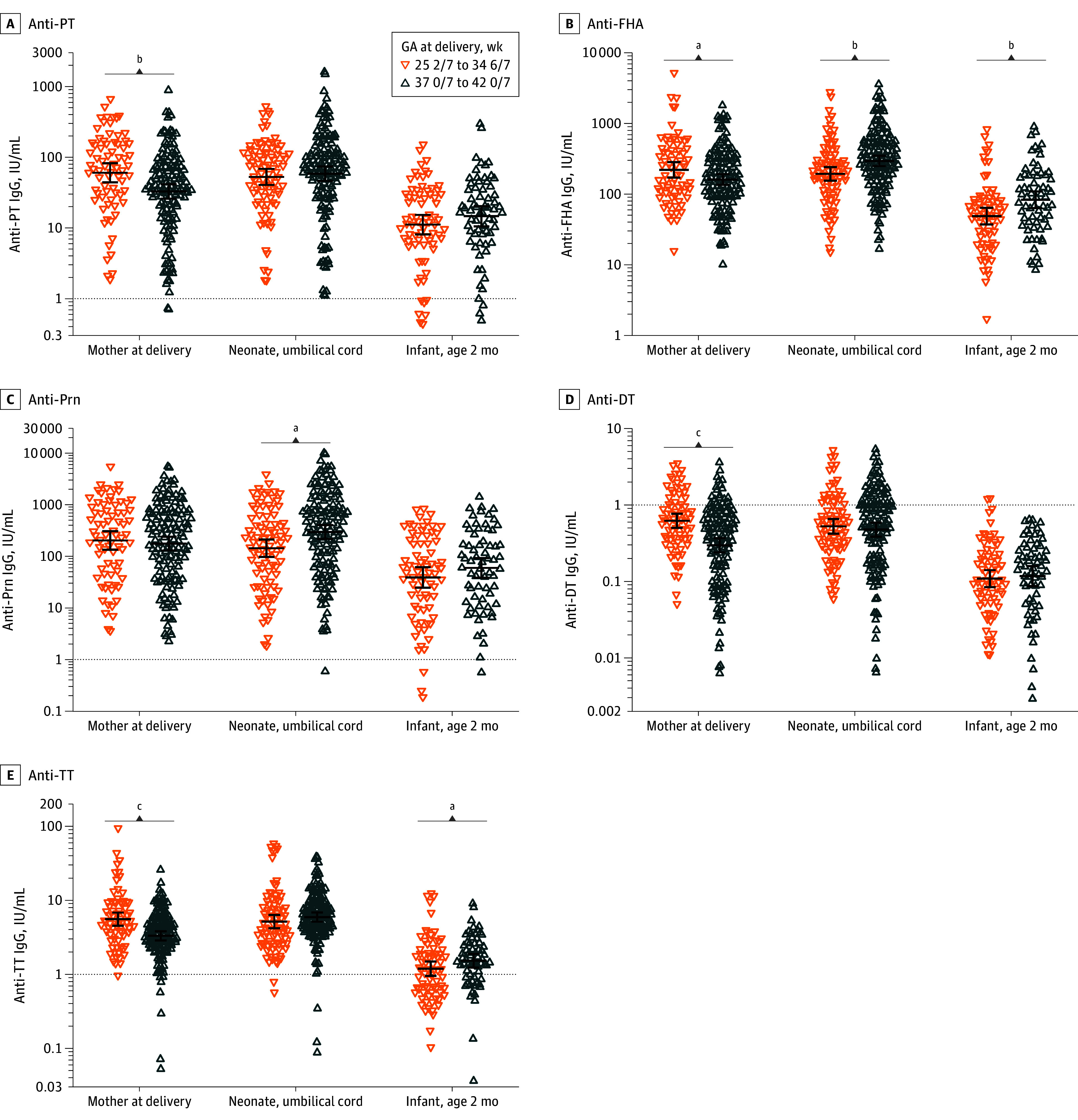
Individual Immunoglobulin G (IgG) Antibody Concentrations and Geometric Mean Concentrations (GMCs) After Second-Trimester Tetanus, Diphtheria, and Pertussis Vaccination in Early- and Full-Term vs Preterm Mother-Infant Pairs at Different Time Points All women were vaccinated between 20 0/7 and 24 0/7 weeks’ gestation (GA). Horizontal lines represent GMCs and vertical bars, 95% CIs. DT indicates diphtheria toxoid; FHA, filamentous hemagglutinin; Prn, pertactin; PT, pertussis toxin; TT, tetanus toxoid. ^a^*P* < .05. ^b^*P* < .01. ^c^*P* < .001.

At 2 months of age after maternal Tdap vaccination between 20 0/7 and 24 0/7 weeks’ gestation, no significant differences in anti-PT GMCs were observed in preterm infants compared with term infants (11.2 IU/mL [95% CI, 8.1-15.3 IU/mL] vs 14.7 IU/mL [95% CI, 10.6-20.4 IU/mL]) (*P* = .23) (Table 2 and Figure 3). In term infants at 2 months of age, besides anti-PT levels, the GMC of IgG against Prn was significantly lower after Tdap vaccination between 20 0/7 and 24 0/7 weeks’ gestation compared with 30 0/7 to 33 0/7 weeks’ gestation (59.8 IU/mL [95% CI, 38.4-93.0 IU/mL] vs 110.3 IU/mL [95% CI, 71.6-170.0 IU/mL]). No differences in GMCs were observed for FHA, DT, or TT for term infants in the study compared with the reference cohort (Table 2 and Figure 2). In preterm infants compared with term infants at age 2 months after maternal Tdap vaccination between 20 0/7 and 24 0/7 weeks’ gestation, GMCs were significantly lower for FHA (48.8 IU/mL [95% CI, 37.3-63.8 IU/mL] vs 83.1 IU/mL [95% CI, 63.6-109.1 IU/mL]) and TT (1.2 IU/mL [95% CI, 1.0-1.5 IU/mL] vs 1.5 IU/mL [95% CI, 1.2-1.9 IU/mL]), whereas no significant differences were observed for Prn and DT (Table 2 and Figure 3).

In umbilical cord serum samples from term offspring, significantly lower GMCs were observed when mothers received Tdap vaccination between 20 0/7 and 24 0/7 weeks’ gestation compared with 30 0/7 and 33 0/7 weeks’ gestation for PT (58.6 IU/mL [95% CI, 46.4-74.2 IU/mL] vs 125.1 IU/mL [95% CI, 94.0-166.3 IU/mL]) and Prn (295.5 IU/mL [95% CI, 216.7-402.8 IU/mL] vs 500.5 IU/mL [95% CI, 322.5-776.7 IU/mL]). No differences were observed for FHA, DT, and TT IgG levels (Table 2 and Figure 2). In umbilical cord serum samples, GMCs from preterm offspring compared with term offspring following maternal Tdap vaccination between 20 0/7 and 24 0/7 weeks’ gestation were significantly lower for FHA (193.5 IU/mL [95% CI, 155.2-241.3 IU/mL] vs 295.2 IU/mL [95% CI, 249.1-349.9 IU/mL]) and Prn (143.7 IU/mL [95% CI, 97.3-212.4 IU/mL] vs 295.5 IU/mL [95% CI, 216.7-402.8 IU/mL]). No differences between term and preterm offspring were observed for PT, DT and TT (Table 2 and Figure 3).

Comparing antibody levels at delivery among mothers of term offspring in the study and reference groups, the anti-PT GMC at delivery was significantly higher in the study group (61.8 IU/mL [95% CI, 46.8-81.7 IU/mL] vs 32.9 IU/mL [95% CI, 26.0-41.6 IU/mL]). No differences for the other Tdap antigens were found (Table 2 and Figure 2). Comparing preterm and term mother-infant pairs following Tdap vaccination between 20 0/7 and 24 0/7 weeks’ gestation, mothers had significantly higher GMCs after preterm than term delivery for all antigens (eg, PT 60.4 IU/mL [95% CI, 44.1-82.7 IU/mL] vs 32.9 IU/mL [95% CI, 26.0-41.6 IU/mL]) except Prn (Table 2 and Figure 3). Sensitivity analyses using a Tdap vaccination cutoff of February 27, 2020, the first day of COVID-19 social distancing measures in the Netherlands, were conducted and found no differences in antibody levels after birth among mothers or infants before vs during COVID-19-measures.

## Discussion

In this prospective cohort study, anti-PT IgG levels in term infants at 2 months of age following maternal Tdap vaccination between 20 0/7 and 24 0/7 weeks’ gestation were inferior to those in the group with Tdap vaccination between 30 0/7 and 33 0/7 weeks’ gestation, with an approximate 2-fold reduction in GMCs of anti-PT IgG levels. As long as the mechanisms of protection following maternal Tdap vaccination are not fully understood and no correlate of protection is available, anti-PT IgG levels are often used in studies like ours as surrogate markers for protection.^18^ Anti-PT levels in umbilical cord blood are correlated with protection against pertussis,^20^ and lower anti-PT IgG levels may point to less protection against pertussis in newborns. We also observed a reduction in anti-Prn antibody levels after maternal Tdap vaccination between 20 and 24 weeks’ gestation compared with the reference group.

The GA at which to administer maternal Tdap vaccination for the highest antibody transfer may vary per vaccine antigen. Many studies have suggested that Tdap vaccination between 27 0/7 and 30 0/7 weeks’ gestation results in maximal pertussis-specific antibody levels and avidity in term offspring, though these studies provided no data on Tdap vaccination before 24 weeks’ gestation.^4,7,21,22,23,24,25,26^ In contrast, an observational Swiss study suggested that Tdap vaccination earlier in pregnancy led to higher maternal antibody transfer, potentially because of the longer transfer time before delivery.^10^ The recent Optimising the Timing of Whooping Cough Immunisation in Mums (OpTIMUM) randomized clinical trial observed the highest pertussis-specific IgG antibody levels in umbilical cord serum when mothers received the Tdap vaccine early in the third trimester (28-32 weeks’ gestation) compared with earlier than 24 weeks’ and 24 to 27 weeks’ gestation.^6^ Notably, the number of preterm offspring included in that study was too small to draw conclusions for this most vulnerable group (15 [4%]; 5 per study group).^6^

The reduction in anti–*B pertussis* antibodies in offspring following maternal Tdap vaccination before 24 weeks’ gestation may possibly be explained by the fact that peak levels of anti–*B pertussis* antibodies following vaccination are achieved when maternofetal antibody transfer is still suboptimal. This appears not to be compensated by the increased time of around 9 weeks for transport until delivery.

Based on epidemiological studies, a minimum of 2 to 4 weeks between maternal Tdap vaccination and delivery seems required for protection of term offspring against clinical pertussis.^6^ Up to 7 to 8 weeks was estimated to result in optimal antibody transfer.^5^ Nevertheless, even in the case of a similar 9-week time interval between maternal Tdap vaccination and delivery in this study, GMCs in preterm offspring following maternal Tdap vaccination between 20 0/7 and 24 0/7 weeks’ gestation were at least 2-fold lower than those in term offspring from the reference group following maternal Tdap vaccination between 30 0/7 and 33 0/7 weeks’ gestation. Also, while a longer interval in case of maternal Tdap vaccination between 20 and 24 weeks’ gestation was associated with almost similar anti-PT antibody levels in term and preterm offspring after a time interval of 9 weeks and 18 weeks, respectively, a significant reduction in anti-PT levels but also anti-FHA and anti-Prn levels compared with Tdap vaccination between 30 0/7 and 33 0/7 weeks’ gestation was observed. The OpTIMUM trial also showed significantly lower anti-PT antibodies after maternal Tdap vaccination at 24 0/7 weeks’ gestation or earlier.^6^

In a study from Belgium,^7^ maternal Tdap vaccination at around 27 weeks’ gestation resulted in improved maternal-derived, pertussis-specific antibody levels in preterm offspring, potentially due to receiving the vaccine at a time in pregnancy with a long enough interval before delivery together with a postvaccination antibody peak during the third trimester that has improved antibody transfer compared with the second trimester. This is relevant for most preterm offspring because currently, 84% of all preterm newborns in the Netherlands are delivered at or after 32 weeks’ gestation.^27^

In the UK, timing of Tdap vaccination changed in 2016 from 28 weeks’ to 16 weeks’ gestation onward. A subsequent analysis of data on pertussis cases in the hospital showed that the effectiveness of maternal Tdap vaccination against pertussis-related hospitalization in infants had remained high.^28^ It must be noted that the overall maternal Tdap vaccination coverage over the period of the study also increased, which might have contributed to robustly high maternal Tdap vaccine effectiveness rates. With an undefined correlate of protection, studies like ours cannot draw conclusions on clinical effectiveness of maternal Tdap vaccination at or before 24 weeks’ gestation. Epidemiological studies on effectiveness with data stratified for GA at birth are required.

While protection against severe pertussis following maternal Tdap vaccination has been confirmed by many observational studies,^8,9^ important knowledge gaps in the protective mechanisms remain. In addition to quantitative antibody levels, quality and functionality of anti–*B pertussis* antibodies may contribute to protection against clinical pertussis, as may maternal immune cells, such as T cells, are transferred to the offspring during pregnancy and may vary with timing of maternal vaccination.^29,30,31^ To our knowledge, the present study is the first to investigate transplacental antibody transfer following maternal Tdap vaccination before 24 0/7 weeks’ gestation in a large group of preterm- and early- to late-term infants up to the age of their first vaccinations and to compare antibody transfer with a well-defined cohort of 55 mother-infant pairs after Tdap vaccination between 30 0/7 and 33 0/7 weeks’ gestation.

### Limitations

This study has limitations. Most importantly, the term offspring from the reference cohort were recruited within different periods. The present study was performed partially during nonpharmaceutical COVID-19 interventions, with reduced *B pertussis* circulation compared with the 2014 to 2016 inclusion period of the reference cohort.^32^ Lower endemic *B pertussis* transmission might result in reduced preexisting antibody levels due to a lack of boosting in women of childbearing age^33^ and may have impacted the antibody response to maternal Tdap vaccination. Sensitivity analyses with a Tdap vaccination cutoff at February 27, 2020, the first day of COVID-19 social distancing measures in the Netherlands, yielded no differences in antibody levels in mothers or infants after birth before and during COVID-19-measures in the present study. The vaccination history of mothers in the study and reference cohorts were similar. Starting in 1957 (ie, the start of the Dutch NIP), a whole-cell pertussis vaccine was used for infant vaccinations. In 1996, an acellular pertussis component was added to the DT-IPV booster dose administered at age 4 years. All participating mothers were born before 2005, the year when the infant acellular pertussis–boosted vaccine replaced infant whole-cell pertussis vaccines. In the near future, more pregnant women will receive the acellular pertussis primary or booster vaccine. This may impact antipertussis immune status and response to maternal Tdap vaccination.^34^

We did not study the antibody response to Tdap vaccination in mothers vaccinated earlier vs later during pregnancy. Potential differences may exist. We found that maternal antibodies at delivery following Tdap vaccination before 24 weeks’ gestation were significantly higher with a shorter interval between Tdap vaccination and delivery, suggesting a rapid decline after peak levels following vaccination. Another limitation is the rate of loss to follow-up for samples. We included more term and preterm infants compared with other studies,^6,7,11^ but many appointments for blood sample obtainment at 2 months of age were cancelled due to COVID-19–related safety measures, resulting in large dropout rates among 2-month-old infants. However, samples from 66 term and 73 preterm infants yielded enough power to assess noninferiority. The dropout group at age 2 months was not selective and not expected to affect results. Finally, we performed a sensitivity analysis to compare mothers with imminent preterm labor recruited on presentation at the hospital with other mothers of preterm offspring vaccinated as part of the study (n = 46 vs 14). We found no statistically significant differences, but differences in clinical baseline factors that may correlate with preterm labor may have occurred. However, we had no clinical data to compare these 2 groups in detail.

## Conclusions

In this cohort study, maternal Tdap vaccination between 20 0/7 and 24 0/7 weeks’ gestation compared with 30 0/7 and 33 0/7 weeks’ gestation was associated with significantly lower anti-PT antibody levels in 2-month-old term and preterm infants despite a similar interval between maternal vaccination and delivery in preterm infants after Tdap vaccination between 20 0/7 and 24 0/7 weeks’ gestation and term infants after 30 0/7 and 33 0/7 weeks’ gestation. Further epidemiological research should determine whether maternal Tdap vaccination before 24 weeks’ gestation provides sufficient protection against clinical pertussis both in term and preterm infants as long as no correlate of protection is available.

## Notes

Supplement 1. **eFigure.** Comparisons for the Primary and Secondary Aim

Supplement 2.
Dutch Maternal Pertussis Vaccine Investigation Group

Supplement 3.
Data Sharing Statement

## References

[1] World Health Organization. Pertussis. 2022. Accessed August 16, 2022. https://www.who.int/health-topics/pertussis

[2] Althouse BM, Scarpino SV. Asymptomatic transmission and the resurgence of *Bordetella pertussis*. BMC Med. 2015;13:146. doi:10.1186/s12916-015-0382-8 26103968 PMC4482312

[3] Malek A, Sager R, Kuhn P, Nicolaides KH, Schneider H. Evolution of maternofetal transport of immunoglobulins during human pregnancy. Am J Reprod Immunol. 1996;36(5):248-255. doi:10.1111/j.1600-0897.1996.tb00172.x 8955500

[4] Healy CM, Rench MA, Swaim LS, . Association between third-trimester Tdap immunization and neonatal pertussis antibody concentration. JAMA. 2018;320(14):1464-1470. doi:10.1001/jama.2018.14298 30304426 PMC6233794

[5] Gomme J, Wanlapakorn N, Ha HTT, Leuridan E, Herzog SA, Maertens K. The impact of timing of pertussis vaccination during pregnancy on infant antibody levels at birth: a multi-country analysis. Front Immunol. 2022;13:913922. doi:10.3389/fimmu.2022.913922 35837400 PMC9273881

[6] Calvert A, Amirthalingam G, Andrews N, ; OpTIMUM Study Group. Optimising the timing of whooping cough immunisation in mums (OpTIMUM) through investigating pertussis vaccination in pregnancy: an open-label, equivalence, randomised controlled trial. Lancet Microbe. 2023;4(5):e300-e308. doi:10.1016/S2666-5247(22)00332-9 37080224

[7] Maertens K, Orije MRP, Herzog SA, . Pertussis immunization during pregnancy: assessment of the role of maternal antibodies on immune responses in term and preterm born infants. Clin Infect Dis. 2022;74(2):189-198. doi:10.1093/cid/ciab424 33971009

[8] Kandeil W, van den Ende C, Bunge EM, Jenkins VA, Ceregido MA, Guignard A. A systematic review of the burden of pertussis disease in infants and the effectiveness of maternal immunization against pertussis. Expert Rev Vaccines. 2020;19(7):621-638. doi:10.1080/14760584.2020.1791092 32772755

[9] Baxter R, Bartlett J, Fireman B, Lewis E, Klein NP. Effectiveness of vaccination during pregnancy to prevent infant pertussis. Pediatrics. 2017;139(5):e20164091. doi:10.1542/peds.2016-4091 28557752

[10] Eberhardt CS, Blanchard-Rohner G, Lemaître B, . Maternal immunization earlier in pregnancy maximizes antibody transfer and expected infant seropositivity against pertussis. Clin Infect Dis. 2016;62(7):829-836. doi:10.1093/cid/ciw027 26797213 PMC4787611

[11] Eberhardt CS, Blanchard-Rohner G, Lemaître B, . Pertussis antibody transfer to preterm neonates after second- versus third-trimester maternal immunization. Clin Infect Dis. 2017;64(8):1129-1132. doi:10.1093/cid/cix046 28329335 PMC5439344

[12] Byrne L, Campbell H, Andrews N, Ribeiro S, Amirthalingam G. Hospitalisation of preterm infants with pertussis in the context of a maternal vaccination programme in England. Arch Dis Child. 2018;103(3):224-229. doi:10.1136/archdischild-2016-311802 28814424

[13] Barug D, Pronk I, van Houten MA, . Maternal pertussis vaccination and its effects on the immune response of infants aged up to 12 months in the Netherlands: an open-label, parallel, randomised controlled trial. Lancet Infect Dis. 2019;19(4):392-401. doi:10.1016/S1473-3099(18)30717-5 30938299

[14] Immink MM, Bekker MN, de Melker HE, Rots NY, Sanders EAM, van der Maas NAT. Study protocol of the PIMPI-project, a cohort study on acceptance, tolerability and immunogenicity of second trimester maternal pertussis immunization in relation to term and preterm infants. BMC Infect Dis. 2021;21(1):897. doi:10.1186/s12879-021-06559-w 34479491 PMC8414744

[15] World Medical Association. World Medical Association Declaration of Helsinki: ethical principles for medical research involving human subjects. JAMA. 2013;310(20):2191-2194. doi:10.1001/jama.2013.281053 24141714

[16] Boostrix. Highlights of prescribing information. October 2023. Accessed October 1, 2023. https://gskpro.com/content/dam/global/hcpportal/en_US/Prescribing_Information/Boostrix/pdf/BOOSTRIX.PDF

[17] van Gageldonk PG, van Schaijk FG, van der Klis FR, Berbers GA. Development and validation of a multiplex immunoassay for the simultaneous determination of serum antibodies to *Bordetella pertussis*, diphtheria and tetanus. J Immunol Methods. 2008;335(1-2):79-89. doi:10.1016/j.jim.2008.02.018 18407287

[18] Callender M, Harvill ET. Maternal vaccination: shaping the neonatal response to pertussis. Front Immunol. 2023;14:1210580. doi:10.3389/fimmu.2023.1210580 37520565 PMC10374427

[19] Højsgaard S, Halekoh U, Yan J. The R package geepack for generalized estimating equations. J Stat Softw. 2006;15(2):11.

[20] Cherry JD. Immunity to pertussis. Clin Infect Dis. 2007;44(10):1278-1279. doi:10.1086/514350 17443463

[21] Abu-Raya B, Forsyth K, Halperin SA, . Vaccination in pregnancy against pertussis: a consensus statement on behalf of the global pertussis initiative. Vaccines (Basel). 2022;10(12):1990. doi:10.3390/vaccines10121990 36560400 PMC9786323

[22] Abu-Raya B, Giles ML, Kollmann TR, Sadarangani M. The effect of timing of tetanus-diphtheria-acellular pertussis vaccine administration in pregnancy on the avidity of pertussis antibodies. Front Immunol. 2019;10:2423. doi:10.3389/fimmu.2019.02423 31681310 PMC6798090

[23] Naidu MA, Muljadi R, Davies-Tuck ML, Wallace EM, Giles ML. The optimal gestation for pertussis vaccination during pregnancy: a prospective cohort study. Am J Obstet Gynecol. 2016;215(2):237.e1-237.e6. doi:10.1016/j.ajog.2016.03.002 26968625

[24] Abu Raya B, Srugo I, Kessel A, . The effect of timing of maternal tetanus, diphtheria, and acellular pertussis (Tdap) immunization during pregnancy on newborn pertussis antibody levels—a prospective study. Vaccine. 2014;32(44):5787-5793. doi:10.1016/j.vaccine.2014.08.038 25173476

[25] Abu Raya B, Bamberger E, Almog M, Peri R, Srugo I, Kessel A. Immunization of pregnant women against pertussis: the effect of timing on antibody avidity. Vaccine. 2015;33(16):1948-1952. doi:10.1016/j.vaccine.2015.02.059 25744227

[26] Abu Raya B, Srugo I, Bamberger E, Kessel A. The avidity of pertussis antibodies following gestational acellular pertussis immunization: reply to Maertens. Vaccine. 2015;33(42):5490-5491. doi:10.1016/j.vaccine.2015.05.090 26071193

[27] Peristat.nl. Perined. 2021. Accessed May 19, 2023. https://www.peristat.nl/

[28] Tessier E, Campbell H, Ribeiro S, . Impact of extending the timing of maternal pertussis vaccination on hospitalized infant pertussis in England, 2014-2018. Clin Infect Dis. 2021;73(9):e2502-e2508. doi:10.1093/cid/ciaa836 32569365 PMC8563224

[29] Cherry JD, Gornbein J, Heininger U, Stehr K. A search for serologic correlates of immunity to *Bordetella pertussis* cough illnesses. Vaccine. 1998;16(20):1901-1906. doi:10.1016/S0264-410X(98)00226-6 9796041

[30] Hellwig SM, Rodriguez ME, Berbers GA, van de Winkel JG, Mooi FR. Crucial role of antibodies to pertactin in *Bordetella pertussis* immunity. J Infect Dis. 2003;188(5):738-742. doi:10.1086/377283 12934190

[31] Marcellini V, Piano Mortari E, Fedele G, ; Pertussis Study Group. Protection against pertussis in humans correlates to elevated serum antibodies and memory B cells. Front Immunol. 2017;8:1158. doi:10.3389/fimmu.2017.01158 28966622 PMC5605623

[32] Middeldorp M, van Lier A, van der Maas N, . Short term impact of the COVID-19 pandemic on incidence of vaccine preventable diseases and participation in routine infant vaccinations in the Netherlands in the period March-September 2020. Vaccine. 2021;39(7):1039-1043. doi:10.1016/j.vaccine.2020.12.080 33478793 PMC7787078

[33] Reicherz F, Golding L, Lavoie PM, Abu-Raya B. Decay of anti-*Bordetella pertussis* antibodies in women of childbearing age following COVID-19 non-pharmaceutical measures. Vaccine. 2022;40(27):3746-3751. doi:10.1016/j.vaccine.2022.04.086 35599039 PMC9091163

[34] Havers FP, Skoff TH, Rench MA, . Maternal tetanus toxoid, reduced diphtheria toxoid, and acellular pertussis vaccination during pregnancy: impact on infant anti-pertussis antibody concentrations by maternal pertussis priming series. Clin Infect Dis. 2023;76(3):e1087-e1093. doi:10.1093/cid/ciac432 35642525

