# Supplementary material for: Maternal Pertussis Immunization and Immunoglobulin G Levels in Early- to Late-Term and Preterm Infants
Source: JAMA Netw Open. 2024 Jul 30;7(7):e2424608. doi: 10.1001/jamanetworkopen.2024.24608 (PMC11289700; doi:10.1001/jamanetworkopen.2024.24608)
Supplement: Supplement 1. — eFigure. Comparisons for the Primary and Secondary Aim [file jamanetwopen-e2424608-s001.pdf]

Supplementary Online Content

Immink MM, Bekker MN, de Melker HE, et a; Dutch Maternal Pertussis Vaccine Investigation Groupl. Maternal pertussis immunization and immunoglobulin G levels in early- and full-term and preterm infants. *JAMA Netw Open*. 2024;7(7):e2424608. doi:10.1001/jamanetworkopen.2024.24608

eFigure. Comparisons for the Primary and Secondary Aim

This supplementary material has been provided by the authors to give readers additional information about their work.

© 2024 Immink MM et al. *JAMA Network Open*.

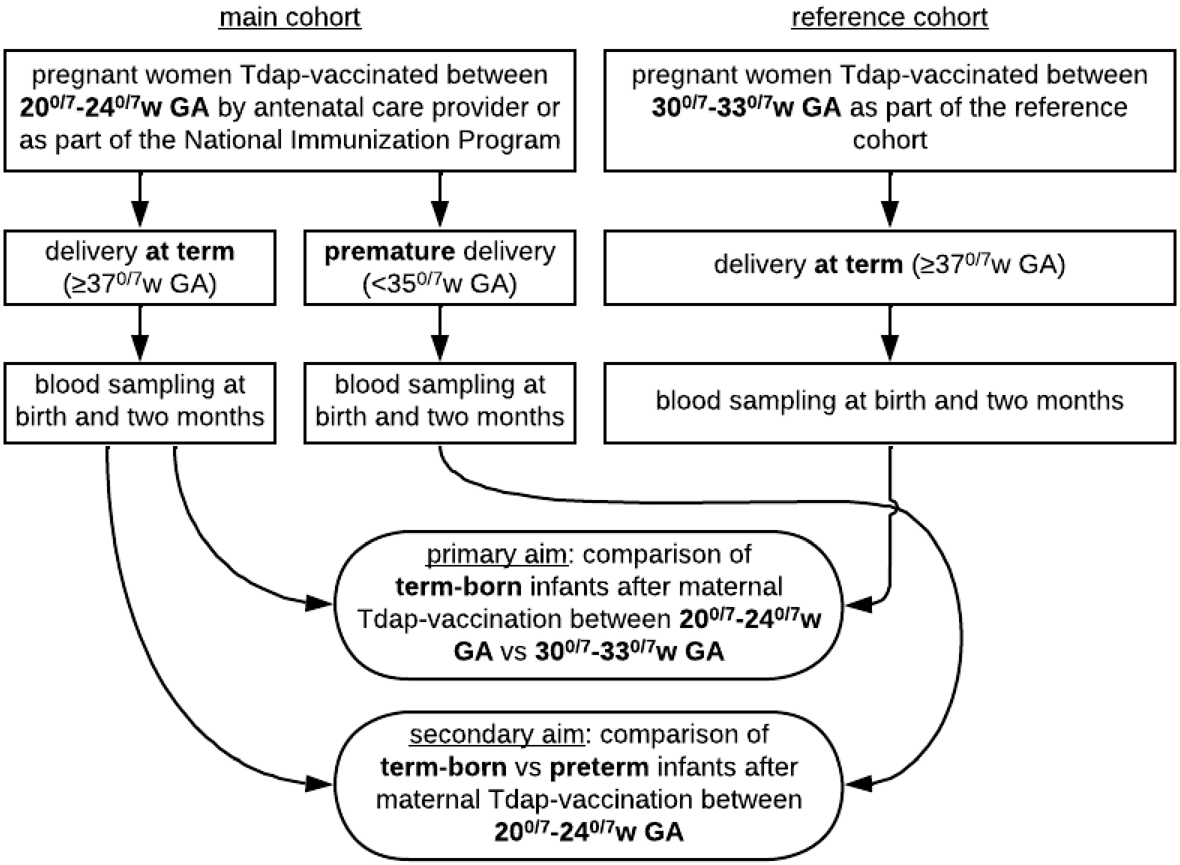

eFigure. Comparisons for the primary and secondary aim.

© 2024 Immink MM et al. *JAMA Network Open*.
