# Supplementary material for: Maternal Pertussis Immunization and Immunoglobulin G Levels in Early- to Late-Term and Preterm Infants
Source: JAMA Netw Open. 2024 Jul 30;7(7):e2424608. doi: 10.1001/jamanetworkopen.2024.24608 (PMC11289700; doi:10.1001/jamanetworkopen.2024.24608)
Supplement: Supplement 2. — Dutch Maternal Pertussis Vaccine Investigation Group [file jamanetwopen-e2424608-s002.pdf]

Supplemental Online Content: Nonauthor Collaborators
*First name, last name, and suffix (if applicable) are required and will appear in PubMed.

*Group Name(s): Dutch Maternal Pertussis Vaccine Investigation Group							
***First Name and Middle Initial(s)**	***Last Name**	***Suffix (eg, Jr, III)**	Academic Degrees	Institution	Location (city, state/province, country)	Role or Contribution, eg, chair, principal investigator	Group (if more than 1 Group listed in the byline) and/or Subgroup (eg, Steering Committee)
Anjoke	Huisjes		MD	department of obstetrics, Gelre Hospital	Apeldoorn, Gelderland, the Netherlands	Principal Investigator (PI) at local hospital	
Kees	Hollander		MD	department of obstetrics, Rivierenland Hospital	Tiel, Gelderland, the Netherlands	PI at local hospital	
Josien	Terwisscha		MD	department of obstetrics, Maasstad Hospital	Rotterdam, Zuid-Holland, the Netherlands	PI at local hospital	
Jek	Persoons		MD	department of obstetrics, Beatrix Hospital	Gorinchem, Zuid-Holland, the Netherlands	PI at local hospital	
Ralph	Scholten		MD	department of obstetrics, Radboud University Medical Center	Nijmegen, Gelderland, the Netherlands	PI at local hospital	
Koen	Deurloo		MD	department of obstetrics, Diakonessen Hospital	Utrecht, Utrecht, the Netherlands	PI at local hospital	
Sander	Galjaard		MD	department of obstetrics, Erasmus University Medical Center	Rotterdam, Zuid-Holland, the Netherlands	PI at local hospital	
Irene	Schiering		MD	department of pediatrics, Spaarne Hospital	Haarlem, Noord-Holland, the Netherlands	PI at local hospital	
