# Supplementary material for: Maternal Pertussis Immunization and Immunoglobulin G Levels in Early- to Late-Term and Preterm Infants
Source: JAMA Netw Open. 2024 Jul 30;7(7):e2424608. doi: 10.1001/jamanetworkopen.2024.24608 (PMC11289700; doi:10.1001/jamanetworkopen.2024.24608)
Supplement: Supplement 3. — Data Sharing Statement [file jamanetwopen-e2424608-s003.pdf]

Data Sharing Statement

Immink. Maternal Pertussis Immunization and Immunoglobulin G Levels in Early- and Full-Term and Preterm Infants. *JAMA Netw Open*. Published July 30, 2024. doi:10.1001/jamanetworkopen.2024.24608

Data
Data available: No

Additional Information
Explanation for why data not available: aggregated anonymized raw data will be available on request
